# Constituents of *Psoralea corylifolia* Fruits and Their Effects on Methicillin-Resistant *Staphylococcus aureus*

**DOI:** 10.3390/molecules200712500

**Published:** 2015-07-09

**Authors:** Yanmei Cui, Shoko Taniguchi, Teruo Kuroda, Tsutomu Hatano

**Affiliations:** 1 Department of Natural Product Chemistry, Okayama University Graduate School of Medicine, Dentistry and Pharmaceutical Sciences, Tsushima-naka, Kita-ku, Okayama 700-8530, Japan; E-Mails: cuiyanmei11@gmail.com (Y.C.); taniguchi@pharm.okayama-u.ac.jp (S.T.); 2 Drug Discovery Technology Center, Okayama University Graduate School of Medicine, Dentistry and Pharmaceutical Sciences, Tsushima-naka, Kita-ku, Okayama 700-8530, Japan; E-Mail: tkuroda@cc.okayama-u.ac.jp

**Keywords:** *Psoralea corylifolia*, bakuisoflavone, bakuflavanone, methicillin-resistant *Staphylococcus aureus*

## Abstract

Two new flavonoids, bakuisoflavone (**1**) and bakuflavanone (**2**), together with 15 known compounds, were isolated from the fruits of *Psoralea corylifolia*, and their structures were characterized by spectroscopic data. The effects of the isolated compounds on methicillin-resistant *Staphylococcus aureus* were also examined. We found that two compounds, isobavachalcone (**10**) and bakuchiol (**12**), showed noticeable antibacterial effects on the MRSA strains examined. Quantitation of the major constituents, including anti-MRSA constituents, was then performed. The results showed individual contents of 1.26%–16.49% (*w*/*w*) among the examined compounds in the ethyl acetate extract from *P. corylifolia* fruits*.*

## 1. Introduction

*Psoralea corylifolia* L. (Fabaceae) is a plant species indigenous to China. Fruits of this plant have been used traditionally for various diseases, including gynecological bleeding, vitiligo and psoriasis. A number of chemical constituents, including flavonoids and coumarins, have been isolated from this plant. Some of these compounds exhibit antioxidant [1], antiplatelet [2], estrogenic [3], immunomodulatory, and antitumor properties [4]. Antibacterial effects of several constituents on *Staphylococcus aureus* and *S. epidermidis* [5] have been reported. A previous report showed that bakuchiol, the main constituent, was also effective on methicillin-resistant *S. aureus* (MRSA) [6].

*Staphylococcus aureus* causes a variety of human diseases, ranging from minor skin infections to severe sepsis, and MRSA has become one of the most frequently encountered antibiotic-resistant bacteria [7]. Several prenylated phenolics obtained from licorice have shown noticeable antibacterial effects on MRSA [8]. Since a number of prenylflavonoids and related compounds were isolated from *P. corylifolia* [5,9,10], this plant species is expected to be a resource of lead compounds for new anti-MRSA drugs.

Our preliminary research showed that *P. corylifolia* fruit extract exhibited remarkable antibacterial effects on MRSA. Therefore, we scrutinized the fruit constituents and isolated 17 compounds, including two new ones, and found that several compounds showed potent antibacterial activity on MRSA. This paper describes the structures of the new compounds, as well as the antibacterial effects of the phenolic constituents on MRSA.

## 2. Results and Discussion

**Figure 1:**
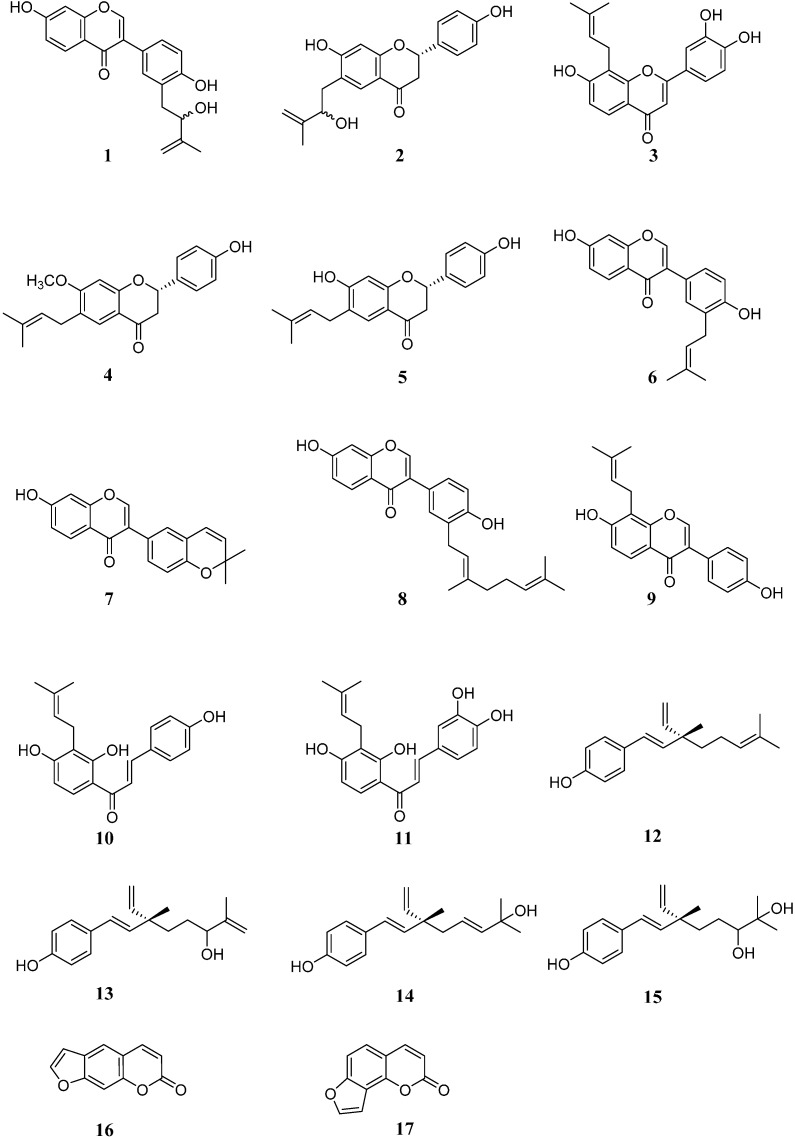
Structures of compounds **1**–**17** isolated from *P.* *corylifolia.*

Column chromatography of the ethyl acetate extract obtained from the fruits of *P. corylifolia* on silica gel, Sephadex LH-20 and MCI gel CHP-20P, and further purification of the fractions by preparative high performance liquid chromatography (HPLC) and thin layer chromatography (TLC), were performed to yield 17 phenolic compounds. Among them, the following 15 were identified as known compounds by comparison of their spectral data with those in the literature: corylifol C (**3**) [5], bavachinin (**4**) [11], bavachin (**5**) [12], neobavaisoflavone (**6**) [11], corylin (**7**) [13], corylifol A (**8**) [5], 8-prenyldaidzin (**9**) [14,15], isobavachalcone (**10**) [11], corylifol B (**11**) [5], bakuchiol (**12**) [16], 3-hydroxy-Δ^1^-bakuchiol (**13**) [16], 2-hydroxy-Δ^3^-bakuchiol (**14**) [16], 12,13-diolbakuchiol (**15**) [17], psoralen (**16**) [18], and isopsoralen (**17**) [19] (Figure 1). The remaining two compounds were regarded as novel and named bakuisoflavone (**1**) and bakuflavanone (**2**) (Figure 1). Their structures were characterized as follows.

### 2.1. Structure Elucidation of the Novel Compounds

Bakuisoflavone (**1**) was obtained as a pale yellow amorphous powder. High-resolution fast-atom bombardment mass spectroscopy (HR-FABMS) revealed the molecular formula C_20_H_19_O_5_ by the [M + H]^+^ ion at *m*/*z* 339.1219. The UV spectrum of **1** showed absorption maxima at 248 and 304 nm, suggesting its isoflavone nature [20]. The ^1^H-NMR spectrum (Figure S1) showed a singlet at δ 8.13, characteristic of the isoflavone H-2 resonance, and signals forming two ABX systems at δ 8.05 (d, *J* = 8.4 Hz, H-5), δ 6.98 (dd, *J* = 2.0, 8.4 Hz, H-6), and δ 6.87 (d, *J* = 2.0 Hz, H-8), and at δ 7.38 (d, *J* = 2.0 Hz, H-2′), δ 7.35 (dd, *J* = 2.0, 8.4 Hz, H-6′), and δ 6.85 (d, *J* = 8.4 Hz, H-5′). These trisubstituted benzene ring signals are assignable to A- and B-ring protons of the isoflavone skeleton, respectively. The spectrum also showed signals corresponding to terminal olefinic methylene protons at δ 4.98 and 4.77 (1H each, br s), an oxygenated methine proton at δ 4.41 (1H, m), a methylene proton at δ 2.90 (2H, m), and protons of a methyl group at δ 1.81 (3H, s), suggesting the presence of a 2-hydroxyl-3-methyl-3-butenyl moiety [21].

**Figure 2:**
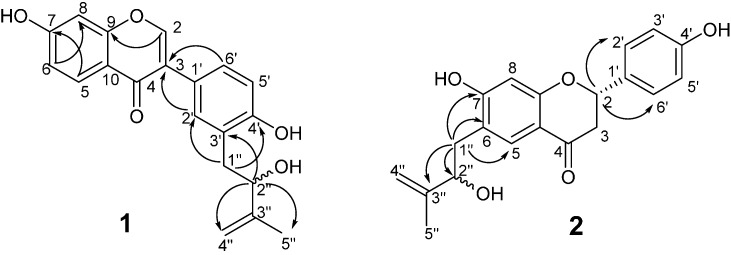
HMBC correlations of compounds **1** and **2**.

The ^13^C-NMR spectrum (Figures S2 and S3) showed 20 carbon signals, corresponding to 15 carbons of the 4′,7-dihydroxy isoflavone structure and to five carbons of the 2-hydroxyl-3-methyl-3-butenyl moiety (Table 1). The HMBC spectrum (Figure S4) showed correlations of H-1′′ of the C5 (2-hydroxy-3-methyl-3-butenyl) moiety to C-2′ and C-4′, and those of H-2′′ to C-3′, indicating the location of the C5 unit at C-3′ on the isoflavone skeleton (Figure 2). The structure of bakuisoflavone (**1**) was thus elucidated to be 4′,7-dihydroxy-3′-(2-hydroxy-3-methyl-3-butenyl)-isoflavone. Since this compound was optically inactive, the formation of an enantiomeric mixture at C-2′′ was assumed.

**Table 1:** ^1^H- and ^13^C-NMR data of compounds **1** and **2**.

No.	1		2	
	δ_H_	δ_C_	δ_H_	δ_C_
2	8.13 (s)	153.1	5.41 (dd, 3.0, 13.2)	80.5, 80.4
3		125.2	3.01 (m)	44.8, 44.7
			2.64 (m)	
4		175.7		190.6
5	8.05 (d, 8.4)	128.5	7.60 (s)	131.4
6	6.98 (dd, 2.0, 8.4)	115.6		121.7, 121.6
7		163.1		164.0
8	6.87 (d, 2.0)	103.1	6.39 (s)	104.3
9		158.7		163.2
10		118.6		114.9
1′		124.1		131.4
2′	7.38 (d, 2.0)	132.9	7.39 (d, 9.0)	128.9
3′		126.6	6.88 (d, 9.0)	116.1
4′		156.8		158.5
5′	6.85 (d, 8.4)	116.7	6.88 (d, 9.0)	116.1
6′	7.35 (dd, 2.0, 8.4)	129.3	7.39 (d, 9.0)	128.9
1′′	2.90 (m)	38.9	2.85 (m)	38.0
2′′	4.41 (m)	77.1	4.38 (m)	76.5, 76.4
3′′		148.5		148.2
4′′	4.98 (s)	110.6	4.95 (s)	110.7
	4.77 (s)		4.77 (s)	
5′′	1.81 (s)	18.3	1.79 (s)	18.4, 18.3

*Note*: 600 MHz, acetone-*d*_6_; chemical shifts in ppm relative to TMS; coupling constants (*J*) in Hz.

Bakuflavanone (**2**), another new compound, was also obtained as a pale yellow amorphous powder. The molecular formula of this compound was assigned C_20_H_21_O_5_, as indicated by the [M + H]^+^ ion at *m*/*z* 341.1383 in the HR-FABMS. The UV spectrum of **2** showed absorption maxima at 235, 274 and 310 nm, suggesting its flavanone nature [22]. Although the ^1^H-NMR spectrum of **2** (Figure S5) showed duplication of the signals, the proton signals at δ 2.64 (H-3), 3.01 (H-3) and 5.41 (H-2) corresponded to the flavanone structure. The A- and B-ring proton signals of the flavanone skeleton were observed as two singlets at δ 7.60 (H-5) and δ 6.39 (H-8) (A ring) and as two 2H signals forming AB doublets (*J* = 8.4 Hz) at δ 7.39 (H-2′, 6′) and δ 6.88 (H-3′, 5′) (B-ring). The spectrum also showed signals representing two terminal olefinic methylene protons at δ 4.98 (1H, br s) and 4.77 (1H, br s), an oxygenated methine proton at δ 4.38 (1H, m), methylene protons at δ 2.85 (2H, m), and methyl protons at δ 1.79 (3H, s), indicating the presence of a 2-hydroxy-3-methyl-3-butenyl moiety in compound **2**.

The ^13^C-NMR spectrum (Figures S6 and S7) revealed 20 carbon signals (15 for the flavanone skeleton and five for the 2-hydroxyl-3-methyl-3-butenyl moiety), although duplication of several signals was also observed (Table 1). In the HMBC spectrum (Figure 2 and Figure S8), correlations between H-1′′ of the C5 (2-hydroxy-3-methyl-3-butenyl moiety) unit to the C-5, C-6, and C-7 signals of the flavanone skeleton indicated that this C5 unit is located at C-6. Bakuflavanone (**2**) was thus characterized to be 4′,7-dihydroxy-6-(2-hydroxy-3-methyl-3-butenyl)-flavanone. The duplication of NMR signals was attributed to racemization at one of the asymmetric centers. The circular dichroism (CD) spectrum of this compound showed a prominent negative Cotton effect at ~306 nm, indicating the *S* configuration at C-2. Therefore, this compound was determined to form a mixture at the C-2′′asymmetric center.

### 2.2. Antibacterial Effects of Isolated Compounds

The antibacterial effects of the compounds isolated from *P. corylifolia* on MRSA were examined by the liquid dilution method [23]. Minimum inhibitory concentrations (MIC) of the tested compounds are shown in Table 2.

#### 2.2.1. Flavone and Flavanones

Among the compounds examined, only one flavone, corylifol (**3**), exerted an antibacterial effect on the two MRSA strains (MIC 16 µg/mL). One of the flavanones, bavachin (**5**), showed a lesser antibacterial effect (MIC 32 µg/mL), while other flavanones, bakuflavanone (**2**) and bavachinin (**4**), did not exhibit antibacterial effects at the concentrations examined.

#### 2.2.2. Isoflavones and Chalcones

Most of the isoflavones showed negligible antibacterial effects (MICs > 32 µg/mL), except for neobavaisoflavone (**6**), which showed an MIC of 16 µg/mL. Two chalcones, isobavachalcone (**10**) and corylifol B (**11**), showed notable antibacterial effects on MRSA (MIC 8 or 16 µg/mL).

#### 2.2.3. Meroterpenes and Coumarins

Bakuchiol (**12**), one of the phenolic meroterpenes, showed a remarkable antibacterial effect (MIC, 8 µg/mL), while the other meroterpenes, **13**–**15**, and coumarins, **16** and **17**, showed negligible effects.

**Table 2:** Antibacterial effects of compounds **1**–**17** against MRSA strains.

Compounds	MIC (μg/mL)	
	MRSA OM481 ^a^	MRSA OM584 ^a^
**Flavone**		
Corylifol C (**3**)	16 (4.7) ^b^	16 (4.7) ^b^
**Flavanones**		
Bakuflavanone (**2**)	>32 (>9.4)	>32 (>9.4)
Bavachinin (**4**)	>32 (>9.5)	32 (9.5)
Bavachin (**5**)	32 (9.9)	32 (9.9)
**Isoflavones**		
Neobavaisoflavone (**6**)	16 (5.0)	16 (5.0)
Corylin (**7**)	>32 (>10)	>32 (>10)
Corylifol A (**8**)	>32 (>8.2)	>32 (>8.2)
8-Prenyldaidzein (**9**)	>32 (>9.9)	>32 (>9.9)
Bakuisoflavone (**1**)	>32 (>9.5)	>32 (>9.5)
**Chalcones**		
Isobavachalcone (**10**)	8 (2.5)	8 (3.1)
Corylifol B (**11**)	16 (4.7)	8 (2.4)
**Meroterpenes**		
Bakuchiol (**12**)	8 (3.1)	8 (3.1)
3-Hydroxy-Δ^1^-bakuchiol (**13**)	>32 (>12)	>32 (>12)
2-Hydroxy-Δ^3^-bakuchiol (**14**)	>32 (>12)	>32 (>12)
12,13-Diolbakuchiol (**15**)	>32 (>11)	>32 (>11)
**Coumarins**		
Psoralen (**16**)	>32 (>17)	>32 (>17)
Isopsoralen (**17**)	>32 (>17)	>32 (>17)

^a^ Clinical isolates from Okayama University Hospital. ^b^ MIC in parentheses is expressed as the unit of 10^−5^ M.

### 2.3. Structure-Activity Relationships

Previous studies have shown that bakuchiol (**12**) and isobavachalcone (**10**) exhibit antibacterial effects on MRSA [24,25], and the present study also showed antibacterial effects of these two on MRSA OM481 and OM584 strains. On the other hand, compounds **13**–**15**, each of which is oxidized at the terminal prenyl group, showed weaker antibacterial effects than that observed for bakuchiol (**12**), which is unoxidized at the terminal prenyl group. Similarly, bakuisoflavone (**1**) and bakuflavanone (**2**), in which the prenyl group is oxidized to 2-hydroxy-3-methyl-3-butenyl group in each structure, showed weaker antibacterial effects than those of the corresponding unoxidized compounds, bavachin (**5**) and neobavaisoflavone (**6**). Therefore, we assumed that hydroxylation of the prenyl group weakened the antibacterial effects. Bavachinin (**4**), in which the hydroxy group at C-7 in bavachin (**5**) is substituted with a methoxyl group, showed a weaker antibacterial effect relative to that of compound **5**. This comparison suggests the importance of the presence of the phenolic hydroxyl group in the flavonoid skeleton.

Based on these observations, we concluded that the presence of both phenolic hydroxy groups and the lipophilicity due to the benzene rings and the prenyl group are required for the potent antibacterial effects on MRSA, and these may contribute to the interaction of the compounds at bacterial cell membranes. Analogous observations have also been reported on the effects of licorice phenolics on MRSA [8] and on vancomycin-resistant *Enterococci* (VRE) [26,27,28].

### 2.4. Quantitative Analysis of the Major Constituents in P. corylifolia Fruit Extract

In the present study, several anti-MRSA constituents were found in *P. corylifolia* fruits, indicating that this plant may be a valuable resource for lead compound development of anti-MRSA drugs. To evaluate the usefulness of this plant, quantitative analysis of the antibacterial constituents was performed. Ethyl acetate extract from *P. corylifolia* was used for HPLC analysis. Quantitation of the major constituents, including anti-MRSA constituents, was based on UV absorption at 280 nm.

**Figure 3:**
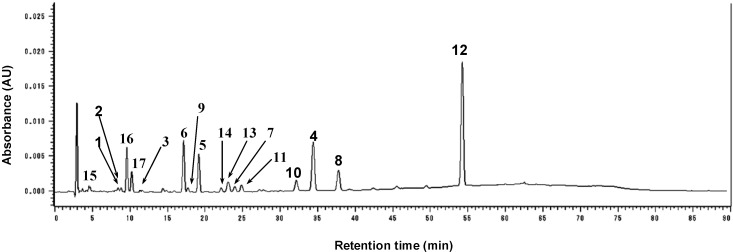
HPLC-UV chromatogram of *P. corylifolia* EtOAc extract at 280 nm ^a,b^. ^a^ Column: YMC-PACK Pro-C18 (6.0 mm i.d. × 150 mm); mobile phase (gradient elution): A, water–acetonitrile–formic acid (60:40:1), B, water–acetonitrile–formic acid (20:80:1); flow rate, 1.0 mL/min; oven temperature, 40 °C; detector, Hitachi L2455. ^b^ The indicated number of each peak is corresponding to the compound number displayed in Figure 1.

The results are summarized in Table 3. Among the peaks caused by constituents (Figure 3), isobavachalcone (**10**) (peak 10) and bakuchiol (**12**) (peak 12) showed especially potent anti-MRSA effects. Among the estimated compounds, bakuchiol (**12**) has the highest ethyl acetate extract content. This observation is in accord with the fact that this compound, a typical meroterpene composed of a phenolic structure and a monoterpene structure, was isolated as the major constituent from *P. corylifolia* [29]. The bavachinin (**4**) content, which is biogenetically derived from bavachin (**5**), was much higher than its mother compound, **5**. The chalcones isobavachalcone (**10**) and corylifol B (**11**) also showed high contents in the extract. These results shown in the present study suggested that the HPLC method can be utilized for quality control with respect to the antibacterial resource.

**Table 3:** Contents of major constituents in *P. corylifolia* EtOAc extract.

Compounds	Content (% *w*/*w*) ^a^
Bavachinin (**4**)	5.03 ± 0.100
Bavachin (**5**)	1.80 ± 0.059
Neobavaisoflavone (**6**)	2.33 ± 0.054
Corylifol A (**8**)	1.86 ± 0.046
Isobavachalcone (**10**)	3.14 ± 0.111
Corylifol B (**11**)	1.81 ± 0.121
Bakuchiol (**12**)	16.49 ± 0.455
Psoralen (**16**)	1.76 ± 0.052
Isopsoralen (**17**)	1.26 ± 0.071

^a^ The value was given as the mean ± standard deviation (SD) based on the triplicate experiments.

## 3. Experimental Section

### 3.1. General Information

^1^H- and ^13^C-NMR spectra were measured on an INOVA AS600 instrument (Varian, Palo Alto, CA, USA, 600 MHz for ^1^H and 151 MHz for ^13^C) in acetone-*d*_6_. Chemical shifts are given in δ values (ppm) based on the chemical shifts of the solvent signals (δ_H_ 2.04, δ_C_ 29.8).

### 3.2. Isolation of Compounds from P. corylifolia

*P. corylifolia* powder (750 g) was extracted in *n*-hexane (3 L × 3), ethyl acetate (3 L × 3) and methanol (3 L × 3), successively. The ethyl acetate extract (22 g) was subjected to silica gel (YMC SIL) (30 mm i.d. × 480 mm) to yield 34 fractions (SIL1-SIL34). SIL5 was identified as bakuchiol (**12**) (576.9 mg). Crystallization of SIL11 and SIL16 from *n*-hexane/EtOAc resulted in psoralen (**16**) (16.5 mg) and isopsoralen (**17**) (57.1 mg), respectively. SIL18 was chromatographed on Sephadex LH-20 and further purified by preparative TLC on silica gel and preparative HPLC on YMC-PACK SIL-A024 (10 mm i.d. × 300 mm) with *n*-hexane/EtOAc (3:1) to yield 3-hydroxy-Δ^1^-bakuchiol (**13**) (15.9 mg) and bavachinin (**4**) (36.9 mg). Column chromatography of SIL20 on silica gel, followed by purification by preparative HPLC on YMC-PACK SIL-A024 (10 mm i.d. × 300 mm) with *n*-hexane/EtOAc (3:1) yielded 2-hydroxy-Δ^3^-bakuchiol (**14**) (13.0 mg). SIL24 was chromatographed repeatedly on YMC SIL followed by Sephadex LH-20 and further purified by preparative HPLC on YMC SIL-A024 (10 mm i.d. × 300 mm) with *n*-hexane/EtOAc (3:1) to give corylin (**7**) (5.0 mg), isobavachalcone (**10**) (19.2 mg) and bavachin (**5**) (21.7 mg). SIL31 and SIL32 were combined and chromatographed on YMC SIL followed by Sephadex LH-20 to give corylifol A (**8**) (11.4 mg) and neobavaisoflavone (**6**) (163.1 mg), respectively. SIL34 was subjected to column chromatography on MCI gel CHP-20P followed by Sephadex LH-20, and the main constituent was further purified by preparative TLC on silica gel followed by preparative HPLC on YMC SIL-A024 with *n*-hexane/EtOAc (3:2) followed by YMC ODS-A324 (10 mm i.d. × 300 mm) with MeOH/H_2_O (7:3) to yield 8-prenyldaidzein (**9**) (22.8 mg), 12,13-diolbakuchiol (**15**) (27.6 mg), corylifol B (**11**) (19.5 mg), corylifol C (**3**) (5.7 mg), bakuisoflavone (**1**) (9.2 mg), and bakuflavanone (**2**) (4.6 mg).

### 3.3. Compound Characterization

*Bakuisoflavone* (**1**): pale yellow amorphous powder. High-resolution FAB-MS: *m*/*z* 339.1219 ([M + H]^+^; calculated for C_20_H_19_O_5_, *m*/*z* 339.1232). UV $\lambda_{\max}^{\mathrm{MeOH}}$ nm (log ε): 248 (4.40) and 304 (2.89). ^1^H- and ^13^C-NMR spectral data (Table 1).

*Bakuflavanone* (**2**): pale yellow amorphous powder. High-resolution FAB-MS *m*/*z*: 341.1383 ([M + H]^+^; calculated for C_20_H_21_O_5_, *m*/*z* 341.1389). UV $\lambda_{\max}^{\mathrm{MeOH}}$ nm (log ε): 213 (4.52), 235 (4.42), 274 (4.25), and 310 (3.91). CD (MeOH) [θ] (nm): +3.31 × 10^4^ (216), +0.23 × 10^4^ (228), +2.01 × 10^4^ (239), −2.36 × 10^4^ (306), and +1.50 × 10^4^ (335). ^1^H- and ^13^C-NMR spectral data (Table 1).

### 3.4. Antibacterial Assay

Two strains of MRSA, OM481 and OM584, clinical isolates from Okayama University Hospital that were stored in a Department of Microbiology laboratory, were used in this study. MICs were estimated by a liquid dilution method. Briefly, tested sample solutions of compounds **1**–**17** were diluted two-fold serially, and pre-cultured bacterial solutions were mixed on 96-well plates and incubated at 37 °C for 24 h. The lowest concentration among the tested samples at which the visible growth was completely inhibited was regarded as the MIC.

### 3.5. Quantitative Analysis of the Major Constituents in P. corylifolia Fruit Extract

Quantitative analysis of the constituents of the *P. corylifolia* fruit extract was carried out on a Hitachi HPLC-DAD D-2000 HSM system (Hitachi, Tokyo, Japan), involving an L-2455 DAD detector, equipped with a YMC-Pack Pro C18 (6.0 mm i.d. × 150 mm) column in an oven at 40 °C. The photodiode array detector was set for obtaining UV absorption from 200 to 400 nm, and the chromatograms at 280 nm were used for the quantitative analyses. The mobile phase was programmed using solvents A [water–acetonitrile–formic acid (60:40:1)] and B [water–acetonitrile–formic acid (20:80:1)] with the gradient mode as follows: 0%–25% B at 0–15 min, 25%–50% B at 15–35 min, 50%–75% B at 35–45 min, 75%–100% B at 45–60 min. The flow rate was set at 1.0 mL/min. Solutions of nine compounds (**4**, **5**, **6**, **8**, **10**, **11**, **12**, **16** and **17**), used as the external standards, were prepared in series (from 0.01 to 1.00 mg/mL) and 2 µL each of the solutions were applied to HPLC, to produce nine individual regression lines to give equations shown in Table 4. Under these HPLC conditions, the relative standard deviation (RSD) 3.7% of the peak area was observed for eight injections of the solution of isopsoralen (**17**). The MeOH solution of the EtOAc extract of *P. corylifolia* fruit (1 mg/mL, 2 µL) was applied to the HPLC analysis, and three independent experiments were conducted for the quantitation of the nine constituents in the extract.

**Table 4:** Parameters for the quantitative analysis of the major constituents of *P. corylifolia* extract.

Compound Used as the External Standard	Retention Time (min)	Range of the Amounts of the External Standard Injected (µg)	Equation of Regression Line for the External Standard *^a^*	*r*^2^ *^b^*
**4**	34.9	0.02–2.00	y = 1.00 × 10^6^x + 1.11 × 10^4^	0.9980
**5**	19.4	0.50–2.00	y = 2.00 × 10^6^x + 0.95 × 10^4^	0.9996
**6**	17.4	0.50–2.00	y = 2.00 × 10^6^x – 0.85 × 10^4^	0.9992
**8**	38.3	0.02–2.00	y = 1.00 × 10^6^x + 0.08 × 10^4^	1.0000
**10**	32.6	0.50–2.00	y = 2.20 × 10^6^x + 0.14 × 10^4^	0.9996
**11**	25.2	0.02–2.00	y = 0.49 × 10^6^x + 0.09 × 10^4^	0.9999
**12**	54.6	0.20–2.00	y = 0.91 × 10^6^x + 0.64 × 10^4^	0.9992
**16**	9.7	0.02–2.00	y = 2.00 × 10^6^x + 1.23 × 10^4^	0.9985
**17**	10.3	0.02–2.00	y = 1.00 × 10^6^x + 0.30 × 10^4^	0.9999

*^a^* The characters x and y represent the amount of the compound injected and the relative peak area shown by the data processor of the HPLC system, respectively. *^b^* Square of correlation coefficient for x and y.

## 4. Conclusions

In the present study, 17 compounds were isolated from the ethyl acetate extract of *P. corylifolia*. Among these compounds, two new compounds, bakuisoflavone (**1**) and bakuflavanone (**2**), were elucidated to be 4′,7-dihydroxy-3′-(2-hydroxy-3-methyl-3-butenyl)-isoflavone and 4′,7-dihydroxy-3′-(2-hydroxy-3-methyl-3-butenyl)-flavanone, respectively. The antibacterial effects of the isolated compounds, which were categorized as a flavone (**3**), flavanones (**2**, **4** and **5**), isoflavones (**1**, **6**, **7**, **8** and **9**), chalcones (**10** and **11**), meroterpenes (**12**, **13**, **14** and **15**), and coumarins (**16** and **17**), were examined. Among them, isobavachalcone (**10**) and bakuchiol (**12**) showed significant anti-MRSA effects. Corylifol C (**3**), neobavaisoflavone (**6**) and corylifol B (**11**) also showed potent antibacterial effects. According to quantitative analysis, these effective compounds are all highly present in *P. corylifolia*. These findings suggest that this plant may be a promising resource for lead compound development of anti-MRSA drugs.

## Supplementary Materials

Supplementary materials can be accessed at: http://www.mdpi.com/1420-3049/20/07/12500/s1.
