# Supplementary material for: Constituents of Psoralea corylifolia Fruits and Their Effects on Methicillin-Resistant Staphylococcus aureus
Source: Molecules. 2015 Jul 9;20(7):12500–11. doi: 10.3390/molecules200712500 (PMC6332258; doi:10.3390/molecules200712500)
Supplement: Supplementary file 1 [file molecules-20-12500-s001.pdf]

Supplementary Materials

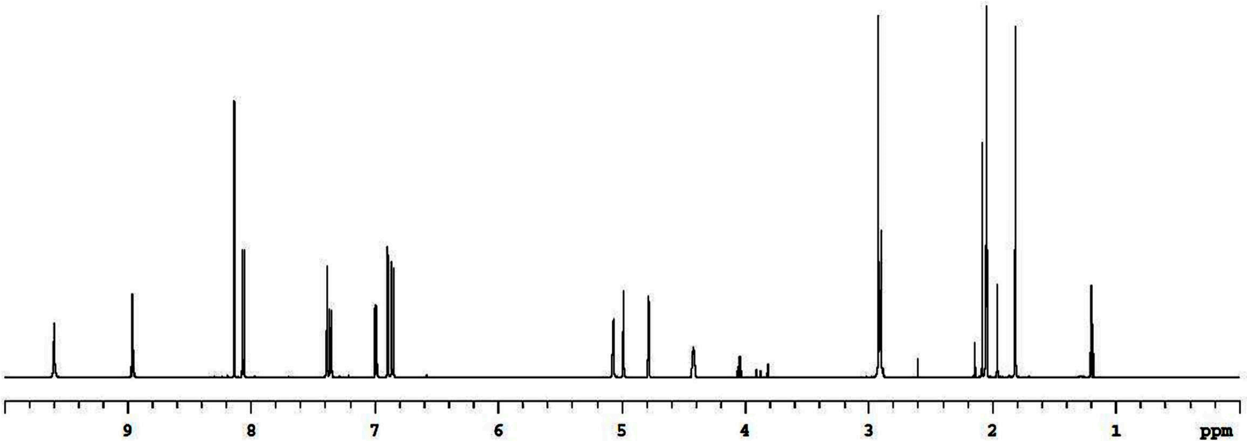

Figure S1. ^{1}H-NMR spectrum of compound **1** (acetone-d_6, 600 MHz).

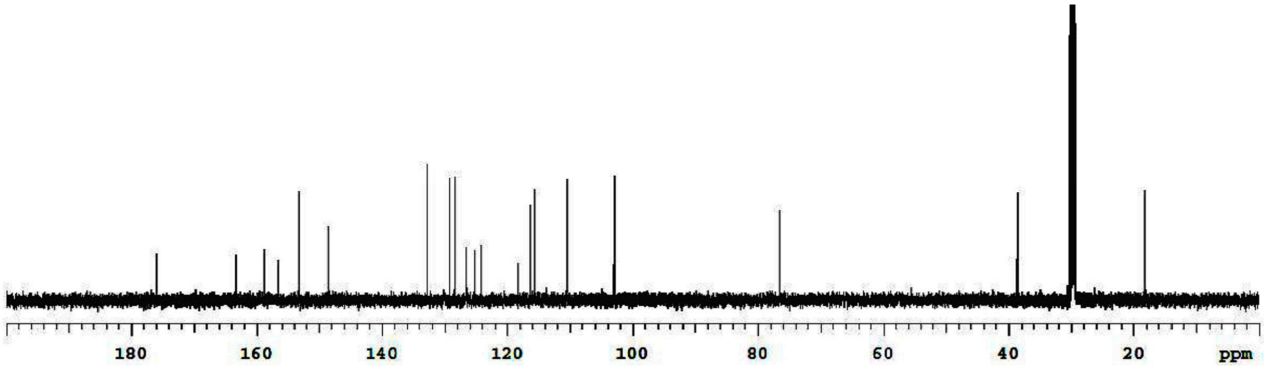

Figure S2. ^{13}C-NMR spectrum of compound **1** (acetone-d_6, 151 MHz).

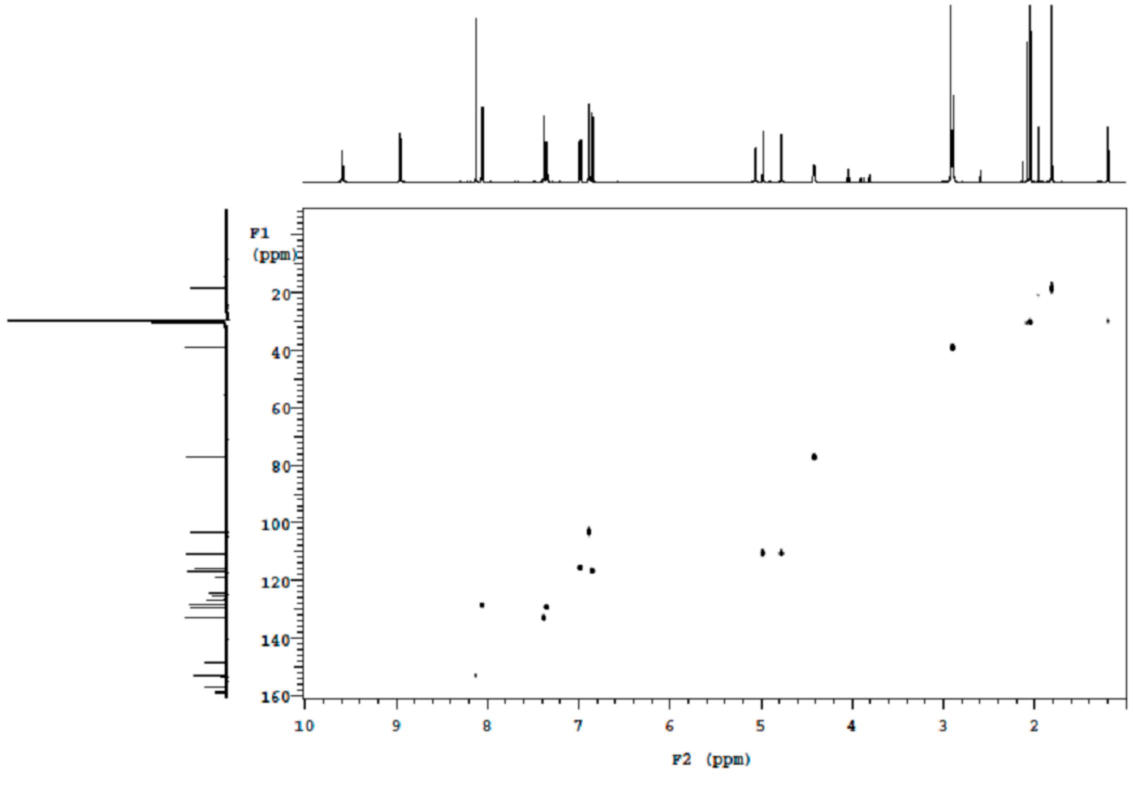

Figure S3. HSQC spectrum of compound **1**.

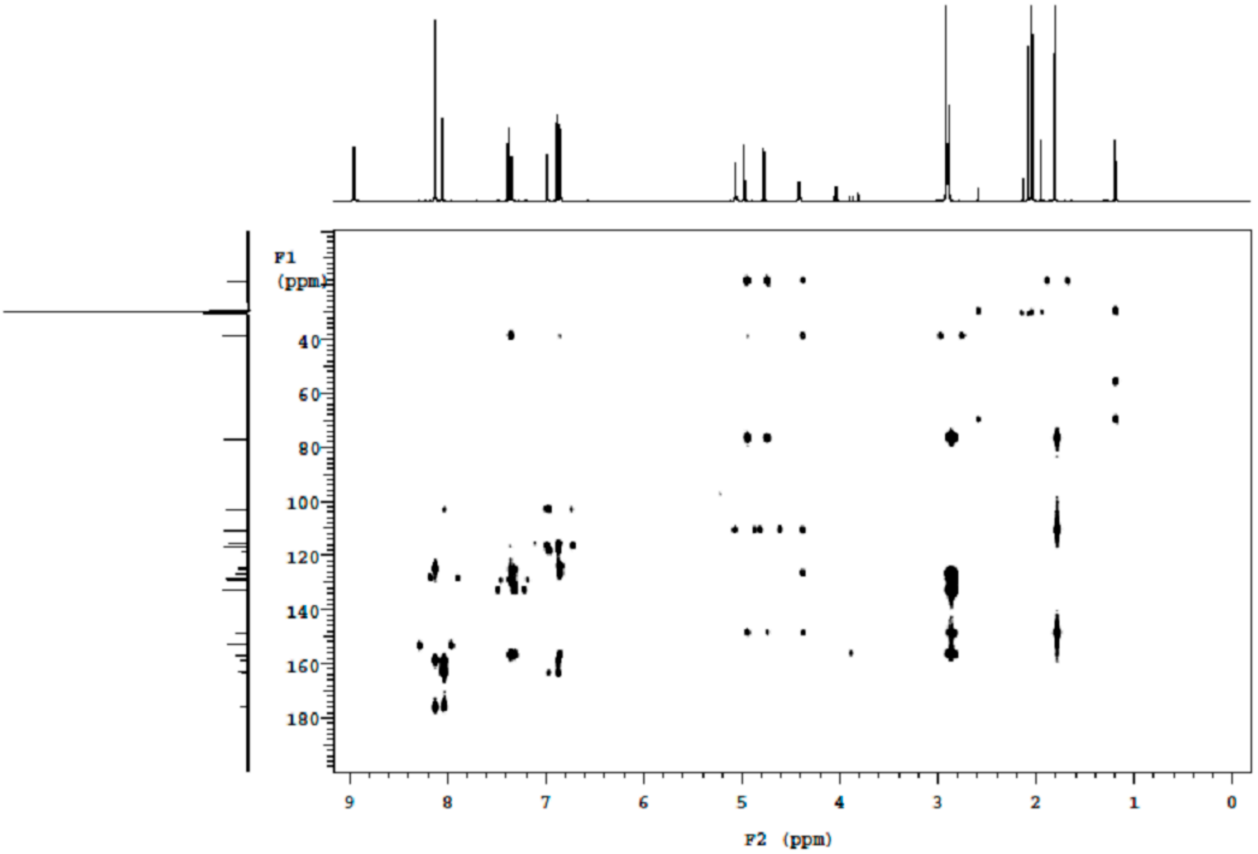

Figure S4. HMBC spectrum of compound **1**.

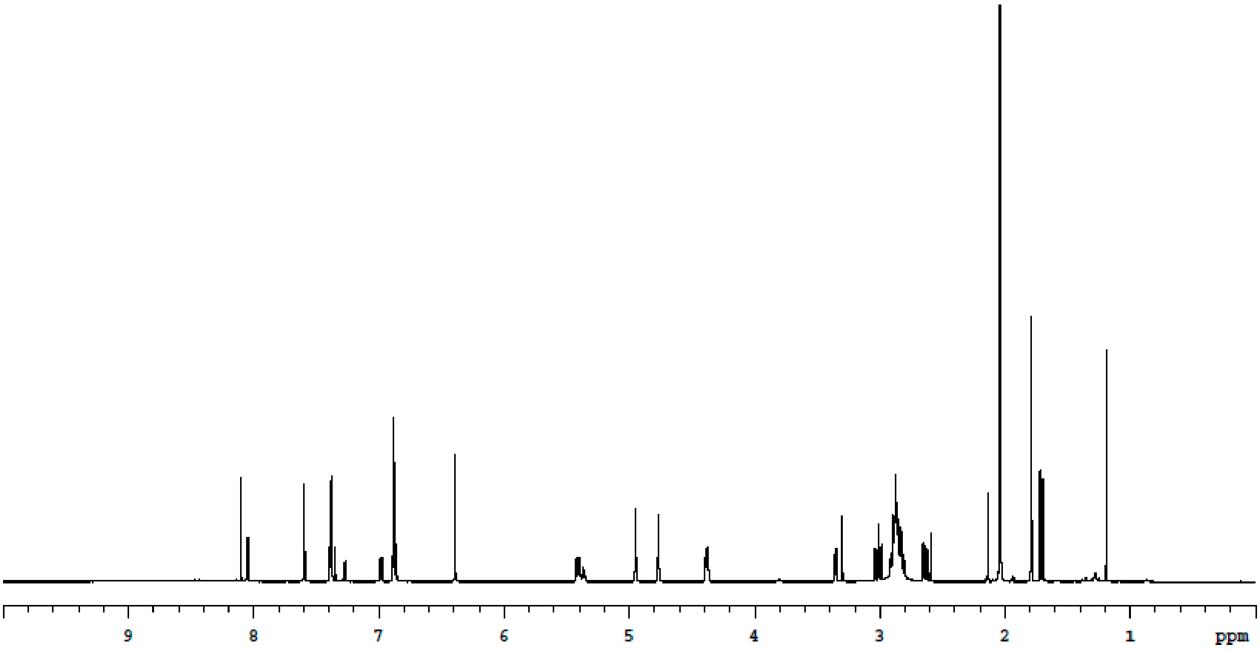

Figure S5. ^{1}H-NMR spectrum of compound **2** (acetone-d_6, 600 MHz).

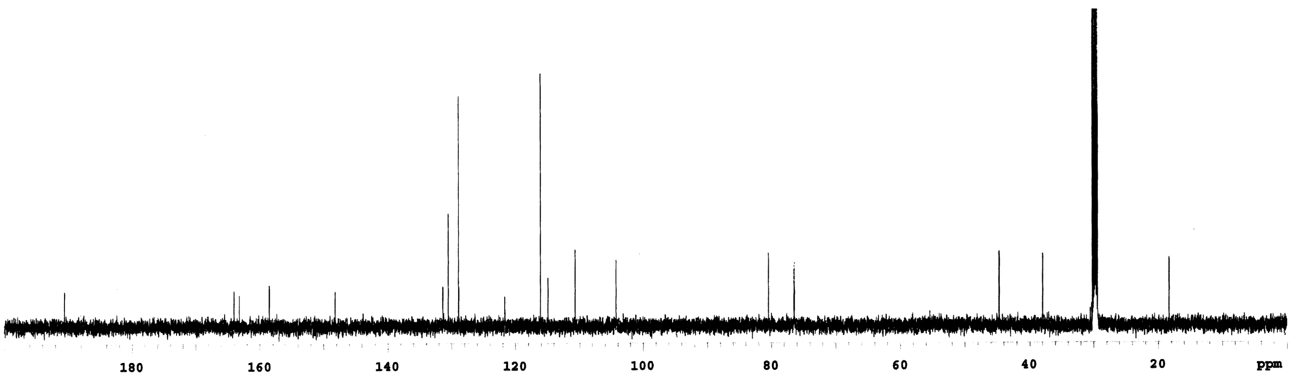

Figure S6. ^{13}C-NMR spectrum of compound **2** (acetone-d_6, 151 MHz).

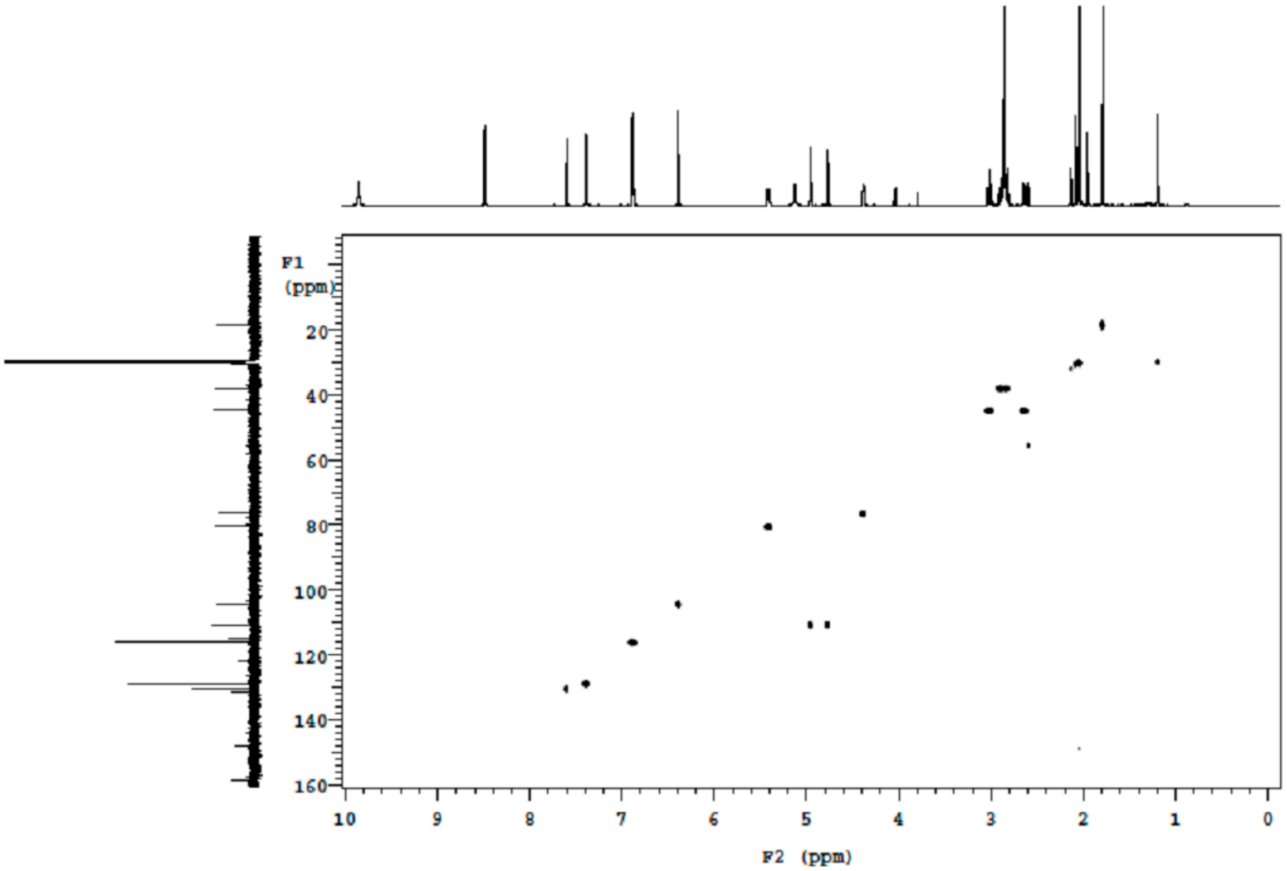

Figure S7. HSQC spectrum of compound **2**.

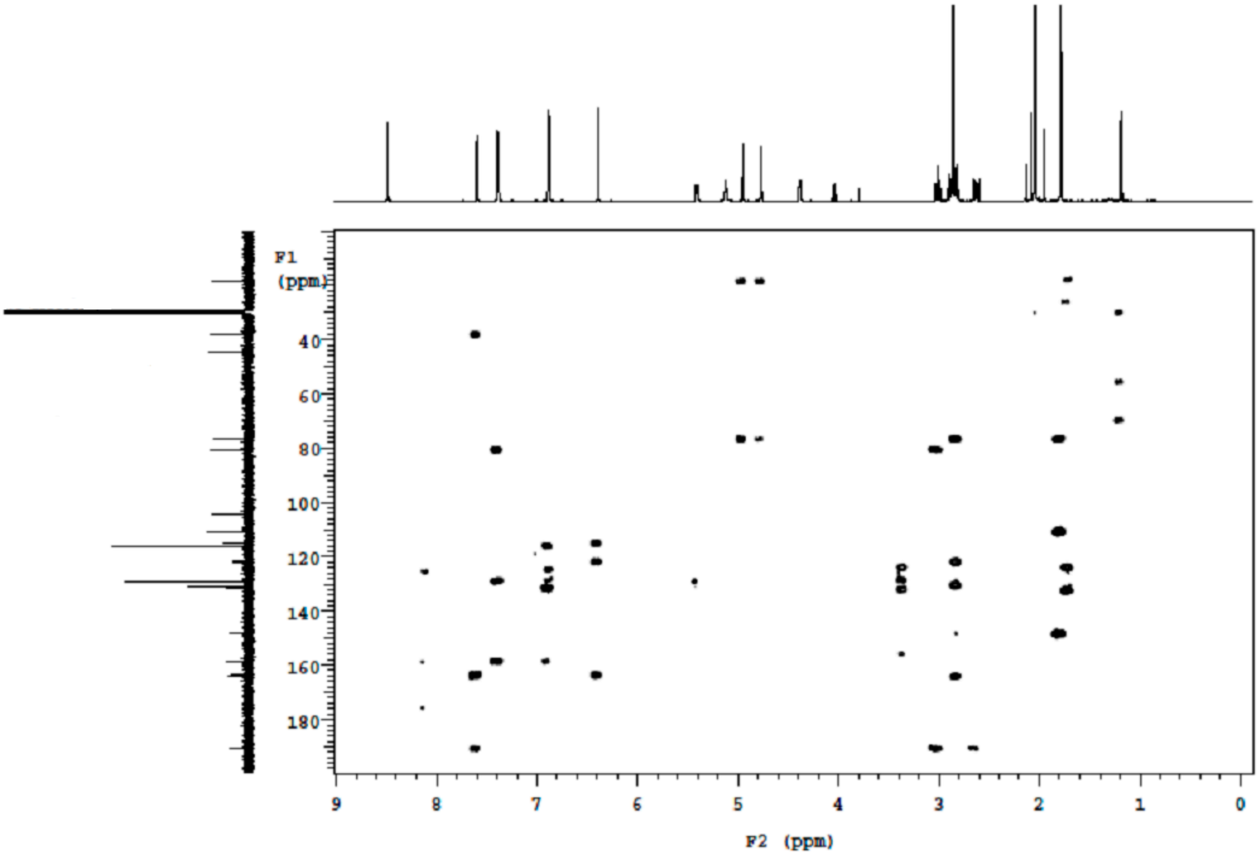

Figure S8. HMBC spectrum of compound **2**.
